# Prognostic Value and Quantitative CT Analysis in RANKL Expression of Spinal GCTB in the Denosumab Era: A Machine Learning Approach

**DOI:** 10.3390/cancers14215201

**Published:** 2022-10-23

**Authors:** Qizheng Wang, Yongye Chen, Siyuan Qin, Xiaoming Liu, Ke Liu, Peijin Xin, Weili Zhao, Huishu Yuan, Ning Lang

**Affiliations:** 1 Department of Radiology, Peking University Third Hospital, Beijing 100191, China; 2 Department of Research and Development, United Imaging Intelligence (Beijing) Co., Ltd., Yongteng North Road, Haidian District, Beijing 100089, China; 3 Beijing United Imaging Research Institute of Intelligent Imaging, Yongteng North Road, Haidian District, Beijing 100089, China

**Keywords:** giant cell tumor of bone, prognosis, tomography, RANKL, radiomics

## Abstract

**Simple Summary:**

Prognostic assessment of giant cell tumor of bone (GCTB) is an ongoing challenge in the treatment and management of bone tumors. Recurrence rates of spinal GCTB are higher compared to GCTB in other bone sites, presumably due to a more aggressive pathology and/or the conservative surgery performed to spare the spinal cord nerve function and decrease postoperative complications. A more accurate prognosis of GCTB will help to inform the choice of treatment methods. This retrospective study investigated prognosis-related molecular markers in spinal GCTB, including RANKL (target of denosumab), focusing on using machine learning analysis based on pre-operative CT to evaluate RANKL status, which may facilitate the selection of better disease management strategies.

**Abstract:**

The receptor activator of the nuclear factor kappa B ligand (RANKL) is the therapeutic target of denosumab. In this study, we evaluated whether radiomics signature and machine learning analysis can predict RANKL status in spinal giant cell tumors of bone (GCTB). This retrospective study consisted of 107 patients, including a training set (*n* = 82) and a validation set (*n* = 25). Kaplan-Meier survival analysis was used to validate the prognostic value of RANKL status. Radiomic feature extraction of three heterogeneous regions (VOI^entire^, VOI^edge^, and VOI^core^) from pretreatment CT were performed. Followed by feature selection using Selected K Best and least absolute shrinkage and selection operator (LASSO) analysis, three classifiers (random forest (RF), support vector machine, and logistic regression) were used to build models. The area under the curve (AUC), accuracy, F1 score, recall, precision, sensitivity, and specificity were used to evaluate the models’ performance. Classification of 75 patients with eligible follow-up based on RANKL status resulted in a significant difference in progression-free survival (*p* = 0.035). VOI^core^-based RF classifier performs best. Using this model, the AUCs for the training and validation cohorts were 0.880 and 0.766, respectively. In conclusion, a machine learning approach based on CT radiomic features could discriminate prognostically significant RANKL status in spinal GCTB, which may ultimately aid clinical decision-making.

## 1. Introduction

Over the last few years, our knowledge of the receptor activator of nuclear factor kappa-B (RANK)/RANK ligand (RANKL) pathway has expanded, and targeting RANKL is being evaluated as anticancer therapy [1,2]. Overexpression of RANKL increases the risk of metastasis in giant cell tumors of bone [3]. However, the effects of RANKL levels on the long-term survival of patients with spinal GCTB have not yet been elucidated.

Denosumab, a monoclonal antibody against RANKL, is a promising treatment option for patients with spinal giant cell tumor of bone (GCTB), and has been approved by the United States Food and Drug Administration (FDA) [4]. Spinal GCTB is usually of the high preoperative stage, and denosumab is used as a targeted therapy for tumors that are difficult to operate upon [5,6]. However, denosumab treatment may lead to extensive recurrence upon treatment withdrawal, and denosumab has been suggested to be used with caution [7,8,9]. Identifying patients who can benefit from denosumab treatment can be helpful in treatment decisions.

The rapid advances in immuno-oncology have revolutionized cancer treatment [10]. As cancer patients respond differently to immunotherapy, the use of targeted drugs is not a blanket statement. An accurate assessment of preoperative RANKL levels may also help clinicians and patients make treatment decisions, including the determination of whether drug therapy, extended resection, postoperative radiotherapy, intensive follow-up, and reasonable clinical expectations. Although tissue biopsy before surgery can be used for disease detection, aside from invasive risk, a small amount of biopsy tissue cannot be used enough to evaluate the entire tumor heterogeneity, and making assessments based solely on small tissue biopsies can lead to bias in diagnosis and evaluation [11].

Imaging is pivotal in assessing tumor heterogeneity. While X-ray is the primary imaging modality for the diagnosis of bone tumors [12], computed tomography (CT) has unique advantages in spinal tumors, and can show the destruction pattern, surrounding invasion, and periosteal reaction pattern of bone lesions without structural overlap. In the era of computer-aided diagnosis, quantitative imaging techniques coupled with machine learning have facilitated the progression of data science research toward clinical translation [13]. Improved imaging methods yielding high-dimensional data enable a more detailed assessment of the macroscopic, cellular, and molecular properties of tumors [14]. Currently, there has been no meaningful research aimed at a non-invasive, comprehensive, and reproducible assessment of RANKL expression levels in spinal GCTB to facilitate predictive decision-making support.

In this study, we evaluate the prognostic role of RANKL status in patients with spinal GCTB, based on postoperative progression-free survival (PFS). In addition, we establish a machine learning model to facilitate the predictive value of RANKL expression levels in spinal GCTB patients. This study demonstrates that the evaluation of RANKL status may support the personalized design of immunotherapy interventions in GCTB.

## 2. Materials and Methods

### 2.1. Patients

This study was approved by the Institutional Research Ethics Board, and adheres to the tenets prescribed by the Declaration of Helsinki (institutional review board “M2019460”, National Clinical Trial number “NCT04952818”). Signed informed consent was waived. We identified 135 consecutive patients with spinal GCTB who underwent surgery in our institution from January 2009 to February 2022. Inclusion criteria were as follows: (a) patients who had a histopathological diagnosis of spinal GCTB; (b) baseline CT performed before surgery, and (c) patients treated by surgical resection.

Among these 135 patients, 28 patients were excluded from this study for the following reasons: (a) The CT scan was performed more than 2 weeks before surgery; (b) Patient had a previous history of biopsy or treatment for the tumor lesion; (c) poor image quality, e.g., with peri-lesion artifacts; (d) missed postoperative paraffin specimen for reanalysis; (e) cases with negative H3F3A (H3 histone, family 3A); and (f) incomplete basic clinical data.

**Figure 1:**
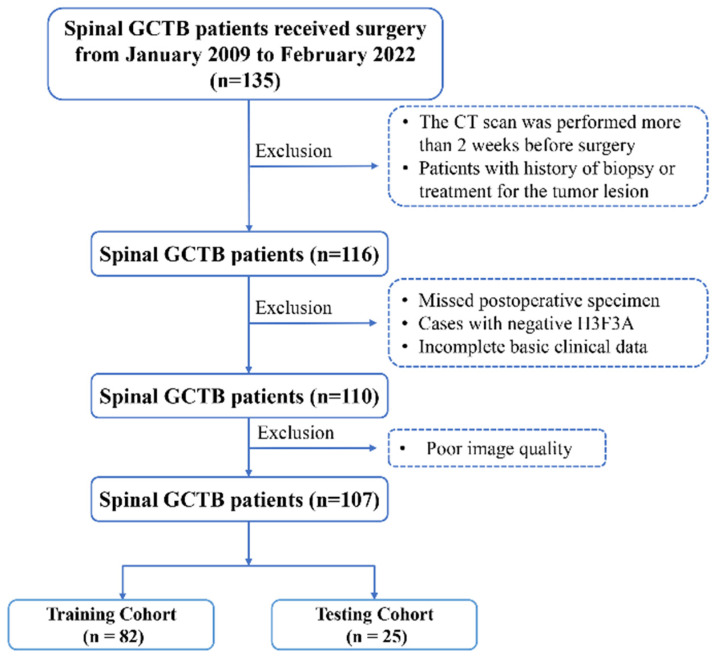
Study flow chart of patient selection. GCTB, giant cell tumor of bone; H3F3A, H3 histone family 3A.

A total of 107 patients (mean age 32.94 ± 12.99 years; M:F = 45:62; age range: 18–71) were included in the final study cohort (Figure 1). Taking September 2019 as the cut-off point, they were divided into a training cohort (*n* = 82) and a validation cohort (*n* = 25).

### 2.2. Immunohistochemistry (IHC) Analysis

Samples from all cases were stained with hematoxylin-eosin (HE) and H3F3A (RevMAb Biosciences, South San Francisco, CA, USA, Catalog No.31-1145-00). IHC staining was performed using an anti-RANKL antibody from Abcam (No. ab222215). From each section, the degree of tumor positivity and the percentage of positive cells were evaluated by two independent pathologists who were blinded to clinical and pathological information. Similar to previous studies [3], RANKL staining was scored based on intensity (0 = no expression; 1 = weak to moderate expression; 2 = strong expression) and percentage of positive cells (0 ≤ 10%; 1 = 10–25%; 2 = 26–50%; 3 ≥ 50%). Cutoff levels for the sum of scores were defined as 0–3 for low expression, and 4–5 for high expression.

### 2.3. Postoperative Follow-Up and Clinical Data

Patients were followed up according to the following guidelines: once every 3 months during the first 2 years; once every 6 months from 3–5 years; and annually after 5 years. Local disease recurrence was confirmed by MRI with an emerging mass found at the resection site with or without a tissue biopsy.

All the clinical data including age, gender, location, tumor stage (Enneking stage), radiotherapy, and denosumab treatment were obtained from the medical records. Simultaneously, imaging assessment of tumor size includes physician measurement (Y) of the longest diameter, while tumor volume is automatically measured by a manually constructed 3D tumor mask.

**Figure 2:**
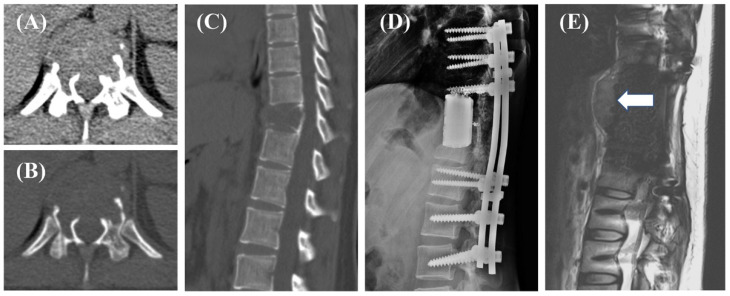
A 29-year-old female, axial (**A**,**B**) and sagittal (**C**) CT images showed the bone destruction of the T12, managed with total en bloc spondylectomy (TES, (**D**)). Postoperative pathology suggested a high expression of RANKL in the tumor. The patient did not undergo radiotherapy. At a 10-month follow-up review, recurrence was detected ((**E**), arrow).

**Figure 3:**
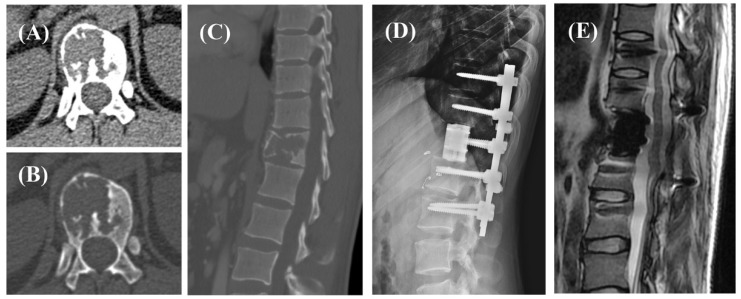
A 32-year-old male, CT images (**A**–**C**) showed a pathologic fracture of the T12 vertebra, managed with TES (**D**). Postoperative pathology showed low expression of RANKL without radiotherapy. At the 32-month follow-up, there was no evidence of recurrence (**E**).

Based on previous studies, surgery and preoperative radiotherapy may have an impact on postoperative recurrence, so we included 75 patients who underwent total en bloc spondylectomy (TES) without preoperative radiotherapy for survival analysis to evaluate the prognostic significance of RANKL levels. Two cases were shown in Figure 2 and Figure 3.

### 2.4. Computed Tomography Imaging and Multiregional Labeling

CT scans were performed using a Sensation-64 scanner (SOMATOM Definition; Siemens, Erlangen, Germany) and a 64-slice spiral CT scanner (Light speed; GE Medical System, London, UK), with parameters including 120 kVp and automatic mAs. The collimator width was 0.625 and 0.60 mm, and the pitch was 1.0. All the CT images were retrieved from the picture archiving and communication system (PACS) in Digital Imaging and Communications in Medicine (DICOM) format. CT scans are isovolumetric and all images can be reconstructed for analysis. 

Three different heterogeneous regions (VOI^entire^, VOI^edge^, and VOI^core^) were segmented and labeled. Manual segmentation framework construction was conducted using Research Portal V1.1 (United Imaging Intelligence, Co., Ltd., Shanghai, China). The VOI^entire^ was segmented on axial CT images by a radiologist with 5 years of experience in spinal tumor diagnoses. Moreover, 35 patients were randomly selected and segmented by another musculoskeletal radiologist with 13 years of experience to construct a test-retest set and calculate the interclass correlation coefficients (ICCs) of radiomic features.

**Figure 4:**
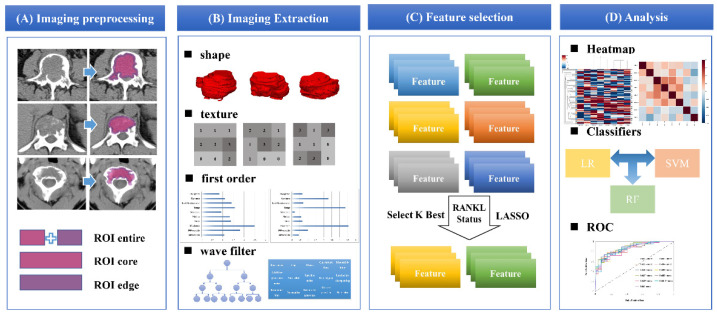
The workflow of necessary steps in this study. (**A**) The VOIs of tumors were manually segmented in axial CT images. Based on the entire tumor VOI, the VOIs of the edge zone and core area were automatically generated. (**B**) The radiomics features were extracted and processed to generate radiomics. (**C**) The sequential feature selection was performed by using Selected K Best and LASSO. (**D**) The RF, LR, and SVM were applied to build the classification models. The performance of the predictive models was evaluated with the area under the ROC curve. VOI, the volume of interest; RF, random forests; LR, logistic regression; SVM, support vector machines; ROC, receiver operating characteristic curve.

Next, VOI^entire^ was shrunk by 2 mm or 3 mm to generate the other two VOIs, the marginal area represents the transition zone of the bone tumor (VOI^edge^), and the remaining core area represents the more central area of the bone tumor (VOI^core^). In detail, VOI shrinkage was performed using the erosion function of the Research Portal V1.1. Figure 4 presents the schema for segmentation, radiomic feature extraction, and predictive modeling. It should be noted that we evaluated the selection of the width of the edge band (VOI^edge^), and the detailed research process is shown in Supplementary Figure S1.

### 2.5. Radiomics Feature Extraction and Preprocessing

Radiomics feature selection, feature extraction, and machine learning models were established on the Research Portal V1.1 (United Imaging Intelligence, Co., Ltd., Shanghai, China). Prior to radiomic feature extraction, B-Spline interpolation resampling was used to normalize voxel size, and anisotropic voxels were resampled to generate isotropic voxels of 1.0 × 1.0 × 1.0 (mm). A total of 2264 radiomics features of each tumor lesion were extracted from preoperative CT imaging, including first-order features, shape features, texture features, and high-level features. The shape features were extracted according to the VOIs in the original image, and the remaining features were extracted in the original image and the filtered image.

Intraclass correlation coefficients (ICCs) were determined to assess the reproducibility of the radiomics features extracted from the VOIs drawn by three independent radiologists. Features with an ICC ≥ 0.8 were considered to be reliable. Before feature selection, all features were normalized by replacing outliers with the median of the particular variance vector and standardizing the data using the Z-score standardization.

### 2.6. Feature Selection and Machine Learning Analysis Strategy

Radiomics features were extracted from different VOIs (multiregions), and then Select K Best analysis and least absolute shrinkage and selection operator (LASSO) methods were used to analyze and screen the extracted image features related to RANKL expression. LASSO regularization involved a parameter, λ, to control the number of selected features; wherein a larger λ retains more features, and the final feature number was determined by λ to maximize the C-index in the training set. A multiple-feature-based radiomics signature (radiomics score, rad-score), was calculated for each patient using a linear combination of features that were each weighted by their respective coefficients.

Three classifiers were used in the prediction models: random forest (RF), support vector machine (SVM), and logistic regression (LR). According to events per independent variable (EPV) values in the multivariable prediction models [15], we selected the 3 features (10 EPV) with the highest efficiency to construct models from the three different heterogeneous regions (VOI^entire^, VOI^edge^, and VOI^core^). At the same time, we compared the model performance of different edge bandwidths of 2 mm and 3 mm. Finally, 15 models were constructed and compared.

### 2.7. Statistical Analysis

Regarding continuous variables, data are expressed as mean ± SD or median interquartile range (IQR). For categorical variables, data are expressed as counts and percentages (*n*, %). The Wilcoxon signed-rank or Kruskal-Wallis tests were used to compare numerical variables, and Fisher’s exact test was used to compare categorical variables. PFS probabilities were estimated using the Kaplan-Meier method and Cox proportional-hazards regression. Statistical analyses were conducted using SPSS (version 24.0, Chicago, IL, USA) and MedCalc (version 15.0, Mariakerke, Belgium). To assess whether the radiomic signature score could separate patients into low or high RANKL expression groups, the area under the curve (AUC) of the receiver operator characteristic (ROC) and its confidence interval (CI) were determined in accordance with the DeLong method. A result was considered statistically significant with a two-tailed *p*-value of *p* < 0.05.

## 3. Results

### 3.1. Patient Information

**Figure 5:**
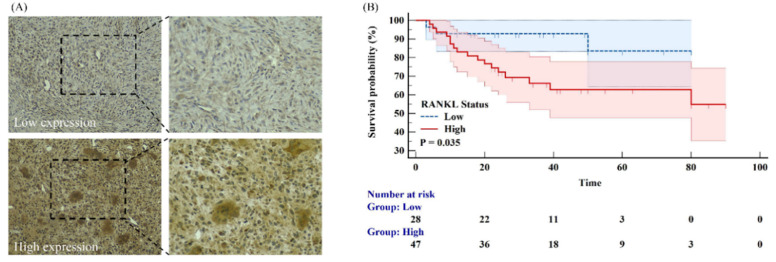
(**A**) Immunohistochemical staining of RANKL in spinal GCTB. Original amplification: 200×. (**B**) Kaplan-Meier survival analysis of the impact of RANKL status.

Of the 107 patients included in the final study cohort (Table 1), sixty-six (62%) spinal GCTB patients exhibited high levels of RANKL expression. The results and grading of immunohistochemical staining are shown in Figure 5A. In the follow-up cohort, RANKL levels were used to indicate a statistically significant difference in PFS, based on Kaplan-Meier survival analysis (Figure 5B). There was no difference in postoperative survival status based on patient age (*p* = 0.190) or gender (*p* = 0.160) (Supplementary Figure S2). No correlation was observed between patient age or gender and RANKL expression (*p >* 0.05), and these factors were not included in the machine learning analysis.

### 3.2. Feature Robustness for Multiregional VOIs

Quantification of all extracted features from each VOI is shown in Supplementary Table S1. The features extracted from the three different VOIs have different numbers of remaining valid features after being screened by the ICC calculation (threshold = 0.8). In the entire tumor vs. margin shrinkage segmentation, the stable feature rates were 51.06% (*n* = 1156), 43.95% (*n* = 995) and 54.37% (*n* = 1231) for VOI^entire^, VOI^edge^, and VOI^core^, respectively. The number of stable features derived from 3D margin shrinkage segmentation was higher compared to the entire tumor and marginal zone (*p* = 0.038). The other groups were not significantly different, with *p*-values of *p* = 0.101 and *p* = 0.200, respectively. Table S1 details the number and percentage of stable features that were obtained with different 3D segmentation methods, grouped according to feature class. In the training cohort, with 10 repetitions of 10-fold cross-validation by the LASSO method, the top 3 features with the most significant coefficients were selected per repetition. Therefore, 300 features with different repetitions were extracted for each VOI. Features and repetitions are shown in Supplementary Figure S3 (edge band width 3 mm). The first three stable and effective features extracted based on each regional VOI were selected for the model construction.

### 3.3. Performance of Models Based on Different Classifiers

**Figure 6:**
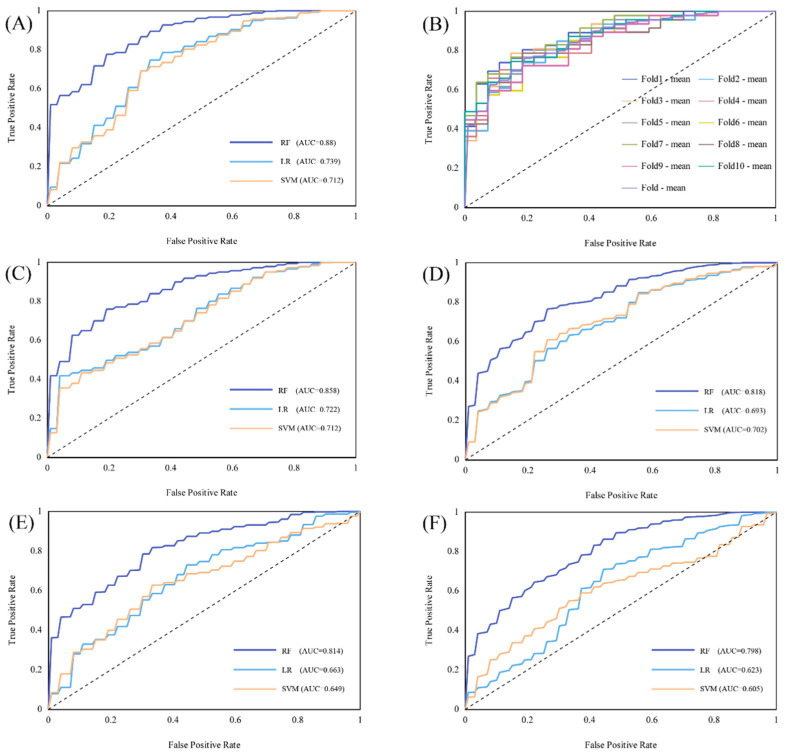
Receiver operating characteristic (ROC) curve demonstrating the performance radiomics models constructed by different classifiers (RF, LR, and SVM) in predicting RANKL status in spinal GCTB based on multiregional VOIs. (**A**) Entire tumor VOI (mean of 10-fold cross-validation); (**B**) Entire tumor VOI (each fold of 10-fold cross-validation); (**C**) Core area VOI (3 mm); (**D**) Edge zone VOI (3 mm); (**E**) Core area VOI (2 mm); (**F**) Edge zone VOI (2 mm). RF, random forests; LR, logistic regression; SVM, support vector machines.

**Figure 7:**
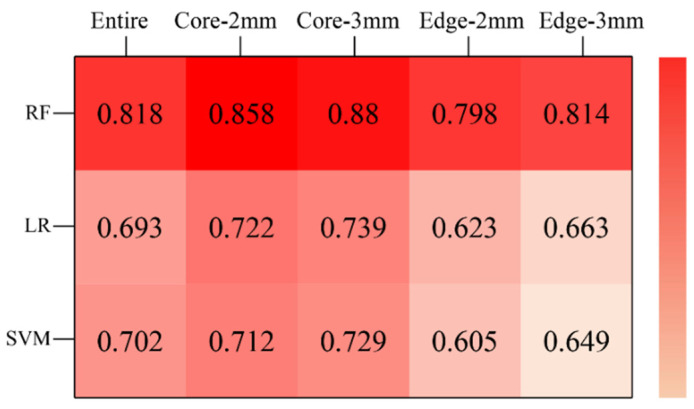
The heatmap of AUCs of three classifiers constructed with five different VOIs. Darker colors mean higher AUC values. AUC, area under the curve; RF, random forests; LR, logistic regression; SVM, support vector machines.

We compared the performance of models based on different VOIs and classifiers (Figure 6). For the radiomics models, the VOI^core^ model was the best performer; the AUC values of the three models are visually displayed in a heatmap diagram (Figure 7). For each VOI model, the performance of the RF classifier was better than that of SVM and LR, and the statistical indicators of DeLong’s test are given in Supplementary Table S2. When the width of the edge band is 3 mm, the model performance of both the core region VOI and the edge region VOI is improved. The corresponding performance parameters of each model (edge width 3 mm), including AUC, F1 score, recall, precision, sensitivity, specificity, and accuracy, are given in Table 2.

### 3.4. Performance and Validation of the Final Prediction Models

**Figure 8:**
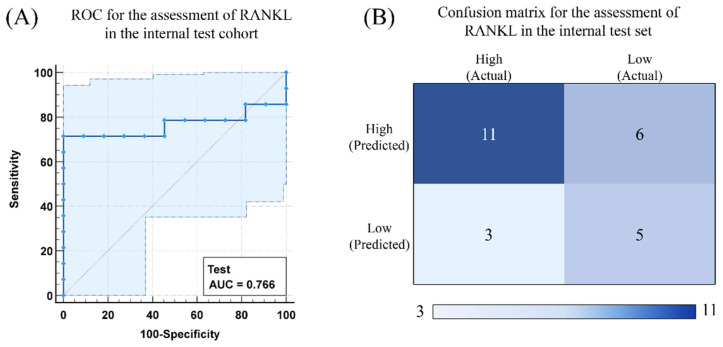
(**A**) ROC curves for prediction of RANKL status in the testing cohorts. (**B**) Confusion matrix for predicted outcomes of RANKL expression levels in the test cohort.

The RF model based on VOIs in the core region with 3 mm edge shrink was selected for independent validation. The features of different VOIs used to build the models are shown in Supplementary Table S3. The ROC curve and confusion matrix of the predicted results are shown in Figure 8. The columns represent the number of predicted classes, and the rows represent the number of true attribution classes of the data. The accuracy, precision, sensitivity, and specificity of the model are 0.640, 0.647, 0.785, and 0.454 respectively.

## 4. Discussion

In this study, we conducted a survival analysis to validate the prognostic value of the RANKL expression levels in a cohort of patients with a postoperative follow-up of spinal GCTB. At the same time, we applied machine learning methods based on radiomics signatures extracted from preoperative CT imaging to classify RANKL status in patients with spinal GCTB.

Treatment of GCTB cases located in the spine is challenging, as en bloc or wide resection is technically difficult and recurrence is common [6]. Improving the prognosis of GCTB of the spine is the focus of surgeons today. Considering the risks faced by patients undergoing the procedure, accurate preoperative stratification of patient prognosis can help in treatment plan planning and precise treatment. Using bioinformatics and combinatorial screening approaches to determine biomarker expression status could be useful in identifying patients who may benefit most from treatment. The RANK/RANKL pathway is often overexpressed and has been positively correlated with tumor progression and advanced disease in primary malignant tumors of the bone, including osteosarcoma, multiple myeloma, and GCTB [16,17,18,19,20]. Moreover, RANK and RANKL expressions are often higher in malignant histological subtypes of bone cancer. For example, RANKL expression is often elevated in Stage III GCTB, and is a useful prognostic marker for predicting the risk of local disease recurrence [21]. Additionally, elevated RANK and RANKL may significantly increase the risk of metastasis [3]. In this study, we reveal for the first time that RANKL expression status is significantly correlated with disease prognosis in patients with spinal GCTB in a clinical cohort. This highlights that drugs targeting the RANK/RANKL pathway may effectively improve patient outcomes beyond merely inhibiting bone tissue destruction. Patients with recurrence with high RANKL expression, as in Figure 2, may be able to improve their prognosis if they are evaluated correctly early preoperatively, are able to suggest drug availability, surgeons expand resection, and postoperative radiation therapy.

Imaging is important in evaluating cancers for surgical planning, prognosis prediction, and post-treatment assessment. Advances in quantitative image analysis methods may offer a more comprehensive approach that includes spatiotemporal information, to produce image-driven biomarkers that may provide a deeper understanding of cancer biology, ultimately aiding in better clinical decisions [22,23]. Campanacci et al. applied conventional lesion size assessment and the new degree of CT ossification in a comparative analysis of 36 patients before and after denosumab treatment. In our cohort, some of the patients treated with denosumab (*n* = 15) partially undergo CT and/or MR scans. It is important to note that despite FDA approval in 2013, denosumab (Xgeva) has been available in our study countries since 2020. Among patients who had CT scans before and after treatment (*n* = 10), only 2 patients had local recurrence, implying that it may be difficult to statistically validate the predictive effect of the new CT evaluation proposed in this study in our cohort. Recurrence is a clinical observation, but it is worth mentioning that this article suggests that tumor treatment response is associated with postoperative recurrence, whereas in terms of microscopic expression, tumor treatment response is associated with therapeutic target status.

Currently, DNA sequencing and immunohistochemistry are the most accurate methods for molecular biomarker assessment in tumor. However, these methods have some limitations such as invasiveness, sampling error, and complications. Tumor multi-omics can be combined with predictive machine learning models, which could be the new digital method on the road to precision cancer medicine. In previous studies of biomarkers for tumor types such as lung cancer, glioma, breast cancer, and prostate cancer, radiomics is found to have the potential as a means to non-invasively predict the status of tumor biomarkers [24,25,26,27,28,29,30,31]. Radiomics approaches combined with a noninvasive machine learning model with tumor immunohistochemistry could improve treatment selection. Our study developed a radiomic signature of RANKL expression status in spinal GCTB based on CT imaging. The optimal model had an AUC of 0.88 in the training cohort, 0.745 in the validation cohort, and 0.766 in the test set. This radiomic signature could assist surgeons in evaluating patient prognosis before surgery and predicting drug treatment response, which would be helpful for clinical decision-making. Previous findings have also applied radiomics for preoperative differential diagnosis, recurrence prediction, and biomarker assessment of GCTB [32,33,34]. Although our study is preliminary, this may be a promising method for improving the precise treatment of spinal GCTB.

This study used three classifiers to construct models based on different VOIs to predict RANKL status in spinal GCTB patients. A total of 15 models were built and compared by ROC analysis. According to the AUCs, the RF classifier was better than the other two classifiers. Previous studies demonstrated the ideal classifiers for the construction of models may differ across organs, and RF may also inform prognostic models for other malignancies, including hepatocellular carcinoma [35], stomach cancer [36,37,38], rectal cancer [39,40], glioma [41], lung cancer [42], pelvic bone tumor [43], and soft tissue tumors [44]. The RF classifier also showed the best performance in recognizing the RANKL status of patients in this study, regardless of the type of VOI.

In radiomics studies, too many features can be selected that do not match the sample size in modeling, which may lead to overfitting. In our study, we follow the 10 EPV rule in binary classification problems [45,46,47]. According to the number of the smallest group in our study cohort (*n* = 30), three features were chosen for the construction of the different models. At the same time, to ensure the robustness of the analysis, we selected the top three most important (in terms of efficacy and frequency) radiomics features with 100 repetitions. This may be a way to improve the repeatability and reproducibility of radiomics. We also summarized the features extracted from multiregional VOIs to compare feature stability. The number of stable features derived from VOI^core^ was higher compared to VOI^entire^, and the features from the VOI^core^ are more concentrated. The RF model based on the tumor core region (VOI^core^) achieved the highest AUC. The study clarified that RF classifiers with the core region of the tumor might perform better in CT image-based GCTB-related tasks.

Some limitations of our study are worth noting. First, this was a single-institution study with a limited sample size, a common concern in “data-hungry” AI-based research. Although our institution is a regionally renowned hospital specializing in spinal tumor surgery, it is quite difficult to expand the sample size due to the incidence of GCTB in the spine. In the future, multicenter studies may improve the robustness and general feasibility of radiomics results. Second, our study did not discuss the effects of surgical procedures on postoperative survival, but only performed survival analysis in patients after TES. TES is the mainstream surgical method for spinal tumors at present, and striving for total vertebral resection is the main consideration of the surgeon unless it cannot be achieved. Third, enhanced CT and MRI images were not analyzed in the retrospective design. In our cohort, preoperative CT without contrast was the most commonly performed (*n* = 107), while relatively fewer patients underwent enhanced CT (*n* = 43) or MRI (*n* = 65). Machine learning attempts based on other scanning modalities may be promising in the future. Finally, due to the limited scope of this study, the generalizability of our findings needs to be confirmed on additional datasets.

## 5. Conclusions

RANKL expression status may be an important molecular marker for the postoperative survival of spinal GCTB patients. We present an RF-based machine learning method that exhibited excellent AUC and stability in assessing RANKL status in patients with spinal GCTB. Radiomics features based on 3D margin shrinkage segmentation showed good performance in the preoperative evaluation of low/high RANKL expression.

## Figures and Tables

**Table 1:** Demographic and clinical information for the study cohort.

Variables	Whole Cohort		
RANKL status	Total	High	Low
	107	66	41
Gender			
Female	62	41	21
Male	45	25	20
Age			
Mean ± SD	32.94 ± 12.99	34.92 ± 13.22	29.76 ± 12.26
Location			
Cervical vertebrae	36	19	17
Thoracic vertebrae	35	18	17
Lumbar vertebrae	26	21	5
Sacrum	10	8	2
Tumor stage			
Stage Ⅱ	40	26	14
Stage Ⅲ	57	40	27
Tumor diameter, cm	4.72 ± 1.99	4.96 ± 1.94	4.34 ± 2.03
Tumor volume, cm^3^	60.75 ± 147.72	54.76 ± 82.81	70.40 ± 215.71
Surgery method			
Partial removal/curettage	32	20	12
Total en bloc spondylectomy	75	45	30
Radiotherapy	22	13	9
Denosumab	15	8	7

**Table 2:** The performance of the RF classifier is based on multiregional VOIs to distinguish RANKL states in the training cohort and validation cohort.

Index	AUC (95%CI)	F1 Score	Recall	Precision	Sensitivity	Specificity	Accuracy
Training cohort							
RF_VOI-entire_	0.814 (0.718–0.912)	0.764	0.720	0.793	0.918	0.581	0.719
RF_VOI-edge_	0.798 (0.695–0.902)	0.810	0.919	0.691	0.979	0.241	0.709
RF_VOI-core_	0.880 (0.807–0.958)	0.857	0.934	0.817	0.720	0.719	0.802
Validation cohort							
RF_VOI-entire_	0.648 (0.419–0.906)	0.674	0.657	0.708	0.657	0.433	0.646
RF_VOI-edge_	0.677 (0.415–0.892)	0.790	0.940	0.688	0.940	0.233	0.682
RF_VOI-core_	0.745 (0.571–0.898)	0.775	0.850	0.728	0.850	0.633	0.694

## Data Availability

Not applicable.

## Supplementary Materials

The following supporting information can be downloaded at: https://www.mdpi.com/article/10.3390/cancers14215201/s1, Figure S1: Two patients with different lesion sizes; Figure S2: Kaplan-Meier survival analysis for gender and age; Figure S3: Feature names extracted from different regions and the number of repetitions. Table S1: The total extracted features of each VOI, the number and percentage of features after ICC inspection of VOIs in different regions; Table S2: Delong test results between different models. Table S3: Features selected by the multi-region VOI models.
