# Supplementary material for: Prognostic Value and Quantitative CT Analysis in RANKL Expression of Spinal GCTB in the Denosumab Era: A Machine Learning Approach
Source: Cancers (Basel). 2022 Oct 23;14(21):5201. doi: 10.3390/cancers14215201 (PMC9658803; doi:10.3390/cancers14215201)
Supplement: Supplementary file 1 [file cancers-14-05201-s001.zip › cancers-1885083-supplementary.pdf]

cancers

MDPI

Supplementary Material: Prognostic Value and Quantitative CT Analysis in RANKL Expression of Spinal GCTB in the Denosumab Era: A Machine Learning Approach

Supplementary Part 1

Research on Edge Band Selection

After VOI was completed for all patients in our cohort, we applied the "erosion" function to preprocess the tumor lesions (VOI^{entire}). Due to the large variation in lesion volume in the study cohort (range: 1105.5-228186.1mm^3, average:10513.0 mm^3), in order to ensure a more reasonable treatment of each lesion, the investigators need to determine the minimum and maximum extent of the marginal zone.

After discussion among researchers, it was agreed that the formation area of 1 mm was too small, especially for larger tumors. On the other hand, it should be noted that excessively large shrinkage criteria will result in loss of core area or very small core area, especially for small lesions. We then tried it on the smallest lesions, 3mm is the maximum acceptable retraction width. Since "erosion" can only take integer values on our operating platform, we next extract the edge band features of 2mm and 3mm, and use the same method to build the models. Both are constructed with 3 features, and the methods are described in the main text of the manuscript.

We selected two patients with different lesion sizes for illustration and explanation (**Figure S1**). The widths of the peripheral strips of different colors are all 1mm, and the colors have no special meaning and are only used for illustration. The first row is a small lesion located in the cervical spine, and the second row is a large lesion located in the sacrum.

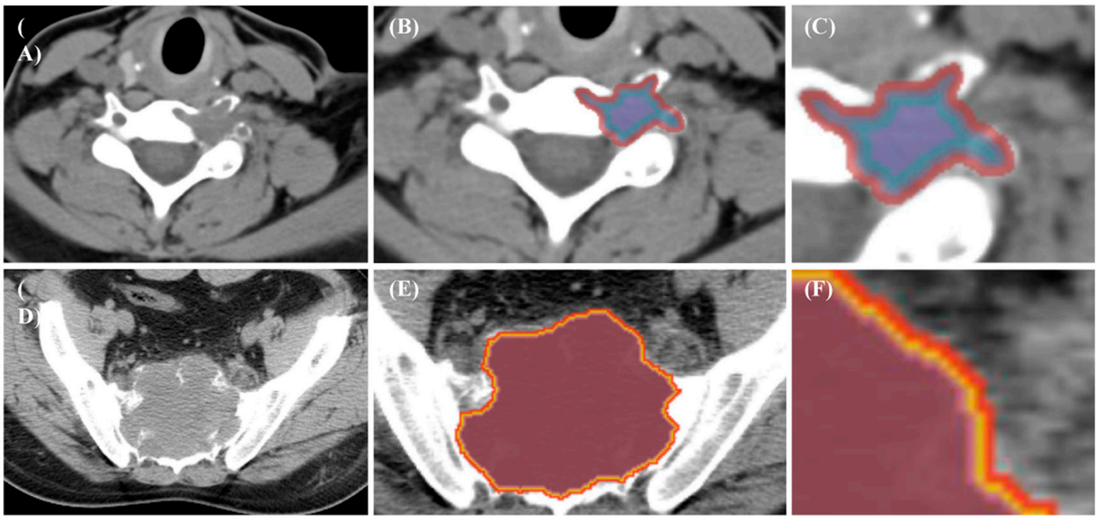

Figure S1. Two patients with different lesion sizes. (**A**–**C**), Cervical spine lesions; (**D**–**F**), sacral lesions. Different color bands at the tumor edge represent 1mm. scale bar or magnification.

Supplementary Part 2

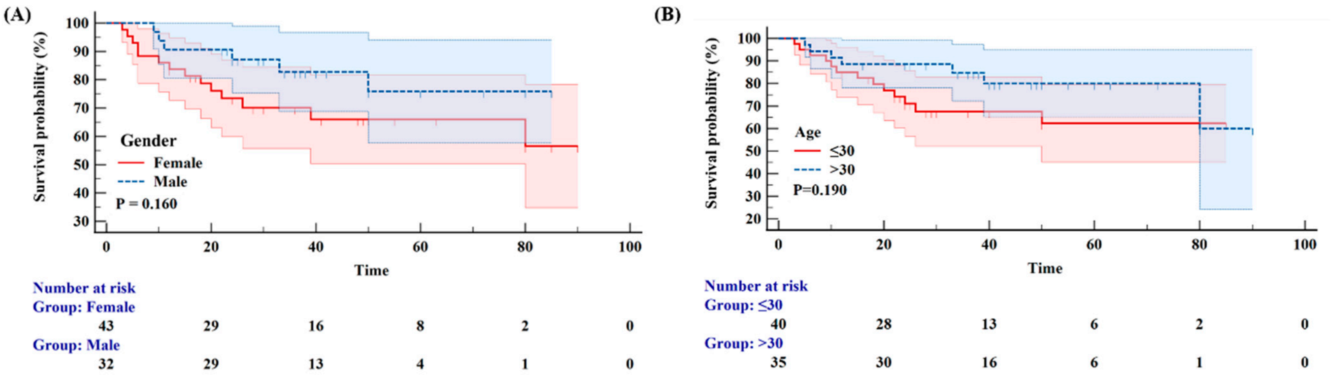

Figure S2. Kaplan-Meier survival analysis for gender (**A**) and age (**B**). The p-values of the two were 0.160 and 0.190, respectively, and there was no statistical difference between the groups.

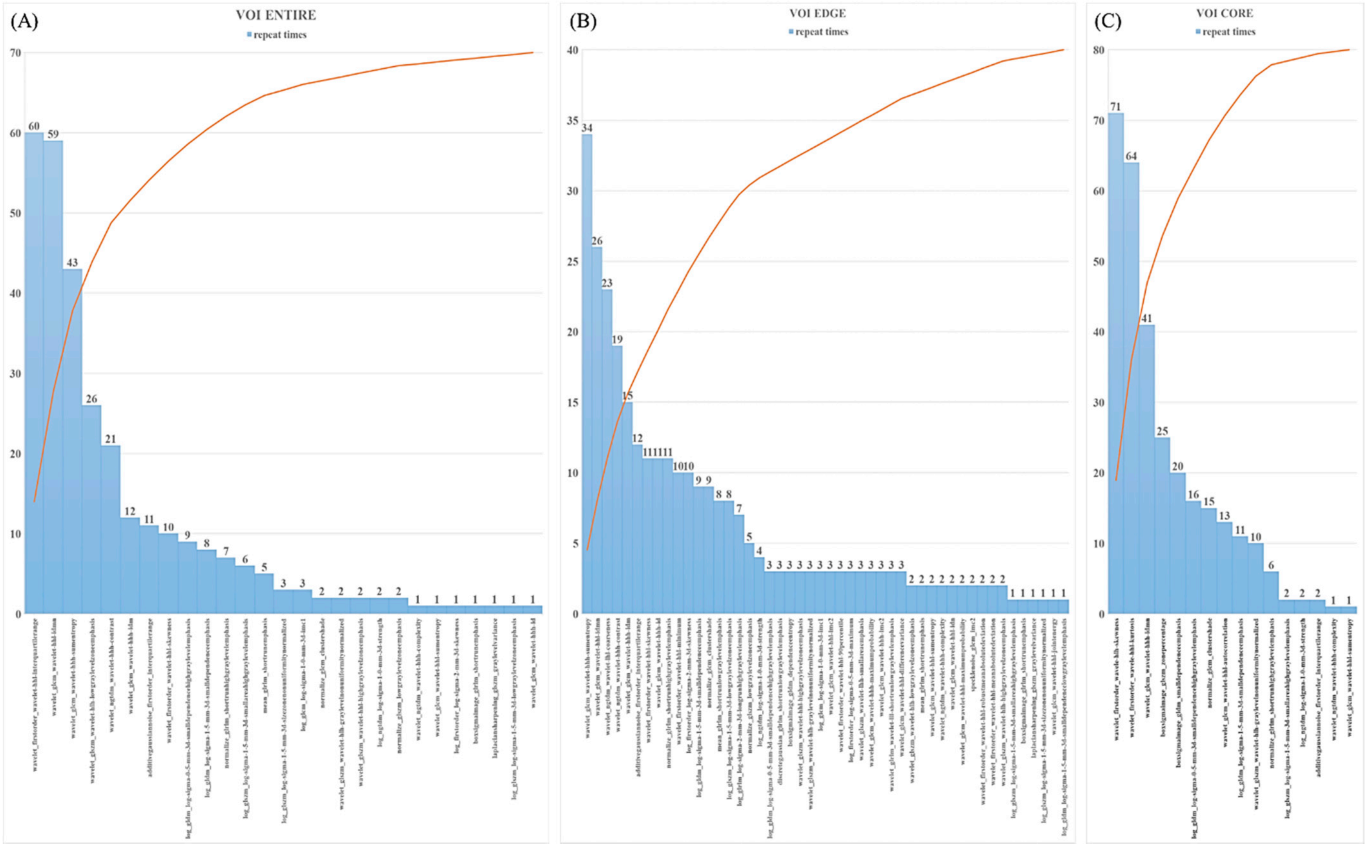

Figure S3. Feature names extracted from different regions and the number of repetitions (top 3 performances for each repetition). The feature naming convention is filter_category_name.

Table S1. The total extracted features of each VOI, the number and percentage of features after ICC inspection of VOIs in different regions (VOI^{entire}, VOI^{edge}, VOI^{core}).

Number / Feature	Total	VOI^{entire}	VOI^{entire} (%)	VOI^{edge}	VOI^{edge} (%)	VOI^{core}	VOI^{core} (%)
**original**	104	87	83.65	79	75.96	94	90.38
**boxmean**	90	51	56.67	40	44.44	53	58.89
**Additive gaussian noise**	90	43	47.78	32	35.56	45	50.00
**binomialblurimage**	90	47	52.22	42	46.67	47	52.22
**curvatureflow**	90	41	45.56	40	44.44	41	45.56
**boxsigmaimage**	90	45	50.00	45	50.00	45	50.00
**log**	360	194	53.89	169	46.94	197	54.72
**wavelet**	720	396	55.00	318	44.17	416	57.78
**normalize**	90	33	36.67	32	35.56	43	47.78
**laplaciansharpening**	90	48	53.33	44	48.89	48	53.33
**Discrete gaussian**	90	39	43.33	31	34.44	39	43.33
**mean**	90	48	53.33	47	52.22	48	53.33
**specklenoise**	90	25	27.78	22	24.44	35	38.89
**recursivegaussian**	90	26	28.89	25	27.78	37	41.11
**shot noise**	90	33	36.67	29	32.22	43	47.78
**Total**	2264	1156	51.06	995	43.95	1231	54.37

Table S2. Delong test results between different models.

	Difference between areas	SE	95% CI	z statistic	Significance
**VOI^{entire}**					
RF vs. LR	0.125	0.0542	0.0293 to 0.242	2.5	0.0124*
RF vs. SVM	0.116	0.0495	0.0322 to 0.226	2.61	0.0091*
LR vs. SVM	0.009	0.0139	-0.0209 to 0.0335	0.454	0.6501
**VOI^{edge}**					
RF vs. LR	0.151	0.0576	0.0392 to 0.265	2.64	0.0083*
RF vs. SVM	0.165	0.0036	0.0718 to 0.204	4.096	< 0.0001*
LR vs. SVM	0.014	0.0645	-0.112 to 0.141	0.225	0.225
**VOI^{core}**					
RF vs. LR	0.151	0.0536	0.0544 to 0.264	2.975	0.0029*
RF vs. SVM	0.141	0.0513	0.0508 to 0.252	2.949	0.0032*
LR vs. SVM	0.010	0.0173	-0.0259 to 0.0420	0.464	0.6423

Note: *, $P < 0.05$. SE, standard error; CI, confidence interval.

Table S3. Features selected by the multi-region VOI models (all features after wavelet filtering).

Multiregions	Feature1	Feature2	Feature3
**VOI^{entire}**	firstorder-hhl-interquartilerange	glcm-hhl-idmn	glcm-hhh-sumentropy
**VOI^{edge}**	glcm-hhl-sumentropy	glcm-hhl-idmn	ngtdm-lhl-coarseness
**VOI^{core}**	firstorder-hlh-skewness	firstorder-hhl-kurtosis	glcm-hhh-idmn
